# Phenotypic Acclimation of Maize Plants Grown under S Deprivation and Implications to Sulfur and Iron Allocation Dynamics

**DOI:** 10.3390/plants11050703

**Published:** 2022-03-06

**Authors:** Filippa S. Maniou, Dimitris L. Bouranis, Yannis E. Ventouris, Styliani N. Chorianopoulou

**Affiliations:** Plant Physiology and Morphology Laboratory, Crop Science Department, Agricultural University of Athens, 75 Iera Odos, 11855 Athens, Greece; bouranis@aua.gr (D.L.B.); yannisventouris@gmail.com (Y.E.V.)

**Keywords:** maize root phenotype, sulfur deficiency, root anatomical traits, sulfur allocation, iron allocation

## Abstract

The aim of this work was to study maize root phenotype under sulfur deficiency stress towards revealing potential correlations between the altered phenotypic traits and the corresponding dry mass, sulfur, and iron allocation within plants at the whole-plant level. The dynamics of root morphological and anatomical traits were monitored. These traits were then correlated with plant foliage traits along with dry mass and sulfur and iron allocation dynamics in the shoot versus root. Plants grown under sulfate deprivation did not seem to invest in new root axes. Crown roots presented anatomical differences in all parameters studied; e.g., more and larger xylem vessels in order to maximize water and nutrient transport in the xylem sap. In the root system of S-deficient plants, a reduced concentration of sulfur was observed, whilst organic sulfur predominated over sulfates. A reduction in total iron concentration was monitored, and differences in its subcellular localization were observed. As expected, S-deprivation negatively affected the total sulfur concentration in the aerial plant part, as well as greatly impacted iron allocation in the foliage. Phenotypic adaptation to sulfur deprivation in maize presented alterations mainly in the root anatomy; towards competent handling of the initial sulfur and the induced iron deficiencies.

## 1. Introduction

Once taken up, a nutrient must be effectively delivered. Delivery involves two processes: (i) part of the uptake is invested in supporting root elongation and growth, maintenance, and activity, whilst (ii) the remainder is transported to the stele. Under deficiency, the delivery process should be performed by minimalizing the delivery cost, reducing the delivery pathways, securing inward movement, and avoiding leakage, as well as the recycling of nutrients from internal pools [1]. Sulfur deficiency has become extensive in many places of the world in recent years. The existence of sulfur deficiency has been reported in cereals in addition to other crops. The reasons for this trend are primarily the large decrease of the atmospheric S deposition and the use of low-S fertilizers [2]. Sulfur is an important nutrient for plants and is believed to be the fourth major plant nutrient after nitrogen, phosphorous, and potassium. [3,4].

The initial uptake of sulfate into the symplast may occur at various sites, possibly through root hairs, in the root periphery, or through the cell walls of the cortex (apoplastic pathway), at a site close to the endodermis, which behaves as an apoplastic barrier [3]. Inside the root symplasm, there may be cell-to-cell removal via plasmodesmata (simplastic pathway). The initial distribution takes place through the xylem. Nonetheless, sulfate is preferably distributed to young developing leaves. Sulfate is redistributed from matured leaves to the root system [4,5], newer leaves [6], or generative sinks (seeds). The vacuoles of mature leaves are a valuable store of sulfur, and redistribution is especially important under sulfur deficiency conditions, even though the effectiveness of this may alter among species.

Sulfur is part of cysteine and methionine, sulfur-containing amino acids with frequent catalytic and structural functions, coenzymes, and prosthetic groups, for instance, iron–sulfur clusters, thiamine, lipoic acid, coenzyme A. These metabolites contain sulfur in the reduced form, notwithstanding, the main form of sulfur available in nature is in the oxidized form of sulfate.

It is also known that after the uptake of SO_4_^2-^ from roots, its reductive assimilation is thought to occur primarily in leaves since the enzymes included in these processes are found in chloroplasts and, to a lower degree, in root plastids [7], even though data on the assimilatory ability of roots is very inadequate. Most of the sulfate reaching leaves is reduced, and reduced S is transported to different sinks, although a portion (10–20%) is often gathered as SO_4_^2−^ [8]. Although sulfate is easily transported from roots and stems, it is not removed but is rather kept in old leaves. These leaves then continue to be green, whereas young leaves turn chlorotic, the typical symptom of sulfur deficiency [9,10]. Earlier experiments have demonstrated that even when sulfur is limited, the growth of plants consisted of 16% of their total sulfur as sulfate, mainly being maintained in the older leaves [4,11]. On the contrary, SO_4_^2−^ accumulated in the roots is freely transported during sulfur starvation.

The main assimilation reactions of the sulfate reduction and the synthesis of cysteine can be outlined as follows: (i) a sulfate activation step, which is catalyzed by ATP sulfurylase (ATPS) to form adenosine 5’-phosphosulfate (APS); (ii) APS reductase (APR), which leads to the formation of sulfite; (iii) Sulfite created by APR is next reduced to sulfide by sulfite reductase (SiR); (iv) Sulfide is thereafter integrated into the amino acid skeleton of O-acetylserine to form cysteine in a reaction catalyzed by O-acetyl serine-(thiol)lyase (OASTL) [12,13].

The assimilatory sulfate reduction pathway can be regulated in at least four manners: (i) by regulating ATP sulfurylase activity, (ii) by the sulfate availability in situ, in place of ATP sulfurylase, (iii) by differences in the level of APS reductase, and (iv) by the 0-acetyl-L-serine availability of cysteine synthase [14,15,16,17].

Iron exists in soils in large quantities, making it the fourth most plentiful element on earth by percentage, after oxygen, silicon, and aluminum. Consequently, the widely restricted availability of Fe for plant nutrition is not associated with its soil content but rather with its limited solubility.

Plant iron uptake has typically been divided into Strategies I and II also called reduction and chelation strategies, respectively [18]. In the rhizosphere, iron is Fe^3+^ mostly represented as oxyhydrates of low solubility. Strategy I is found in non-graminaceous monocots and dicots. It relies on the reduction of Fe^3+^ by a ferric chelate reductase and the uptake of the resulting Fe^2+^ into the root cells through iron-regulated transporters (IRTs). Strategy II is found in grasses. Maize belongs to the grass family (Poaceae) and represents a Strategy II plant, which excretes phytosiderophores (PS), described as plant-derived slight, organic molecules with a large affinity to iron [19]. The resulting Fe^3+^-PS complexes are carried to the roots by the oligopeptide transporter YS1, initially identified in maize [20].

As a consequence of its toxicity and low solubility, iron must form complexes with chelates to be transferred successfully without creating harmful redox reactions. [21]. Physiological and molecular research has shown some major chelators in the plant body, for instance, citrate and nicotianamine [22,23]. Fe^3+^-citrate is the dominant form of iron found in xylem secretions, and citrate is believed to be associated with long-distance iron transportation from roots to shoots [24]. Nicotianamine is a non-protein amino acid that forms chelates with both Fe^2+^ and Fe^3+^ and is the precursor of phytosiderophores [22,23]. Nicotianamine is constructionally comparable to phytosiderophores and chelates iron for intercellular transportation into the phloem. Iron is essential for photosynthetic electron transport, chlorophyll biosynthesis, Fe–S cluster formation, heme biosynthesis, and other vital metabolic processes that take place in chloroplasts [25]. In plants, ferritin, an iron storage protein, is found in plastids [26]. Mitochondria are organelles with a high iron requirement [25]. Iron is utilized as a cofactor in the respiratory electron transport chain, and Fe–S clusters are concentrated in mitochondria besides chloroplasts.

The interaction between iron and sulfur nutritional status’ might be of special significance because the majority of the metabolically effective Fe is linked to S in Fe–S clusters. The biosynthesis of Fe–S clusters demands the supply of reduced S and chelate Fe at a determined stoichiometric proportion, strongly indicating cooperation between the metabolisms of the two nutrients. [27]. Nutrition starvation experiments with barley also revealed a positive correlation between the plant’s sulfur nutritional status and its ability to confront Fe deficiency. One of the first reactions to Fe deprivation in the family Poaceae is the extrusion of PSs into the rhizosphere for Fe^3+^ chelation and solubilization [28]. Phytosiderophores are synthesized from nicotianamine, the precursor of which is S-adenosyl-methionine, thereby describing another possible connection between Fe and S metabolism. Under sulfate deprivation, the release of PSs was decreased, whereas when barley plants were re-supplied with sulfate, the release of PSs was enhanced [29,30]. A significant decrease in iron accumulation caused by S deficiency has been noticed in durum wheat [31] as well as in rice plants [32]. The complicated regulation of both iron and sulfur homeostasis contains various regulating pathways with probable dissimilar signal molecules [33]. This complexity is primarily based on the subsequent perspectives: (i) different adjusting mechanisms could operate locally (cell and/or tissue) and systemically (shoot-root communication); and (ii) plant responses to nutrient deficiency are adapted to the degree of stress severity.

The objective of the present work was to study maize root phenotype under sulfur deficiency stress, to reveal the correlations between the altered phenotypic traits and the corresponding dry mass, sulfur, and iron allocation within plants at the whole plant level. In line with this, several root morphological traits related to nutrient exploration and uptake (i.e., root types, root length, root sectors, number and length of lateral roots), as well as anatomical traits related to the transport of nutrients to the aerial plant parts (i.e., the cross-sectional areas of epidermis, cortex, hypodermis, aerenchyma, central cylinder, along with number, cross-sectional area, and volume of metaxylem vessels) were monitored. These traits were then correlated with plant foliage (i.e., laminas and sheaths, length, and surface area of laminas), along with dry mass, sulfur (sulfate vs. organic sulfur), and iron allocation dynamics, in the shoot vs. the root.

## 2. Results

### 2.1. Plasticity of Phenotypic Traits

#### 2.1.1. Root Morphological Features Related to Nutrient Exploration and Uptake

On d0, only the embryonic root system had formed, consisting of two root types: the primary root (PR) and several seminal roots (SR). On d10 under full nutrition, in addition to the embryonic root system, two other root types had emerged, the mesocotyl roots (MR) and the first group of crown roots (CR1), whilst on d19, the second and third groups of crown roots were added to the root system (CR2, CR3).

On d10 under sulfate deprivation, the length of each root type was comparable to its counterpart in the control (C) plants. The length of the LR sector of PR and MR was longer than that of control plants. The deprivation increased the number of SR and MR. Both the PR and SR had more but shorter lateral roots compared to control plants.

**Figure 1:**
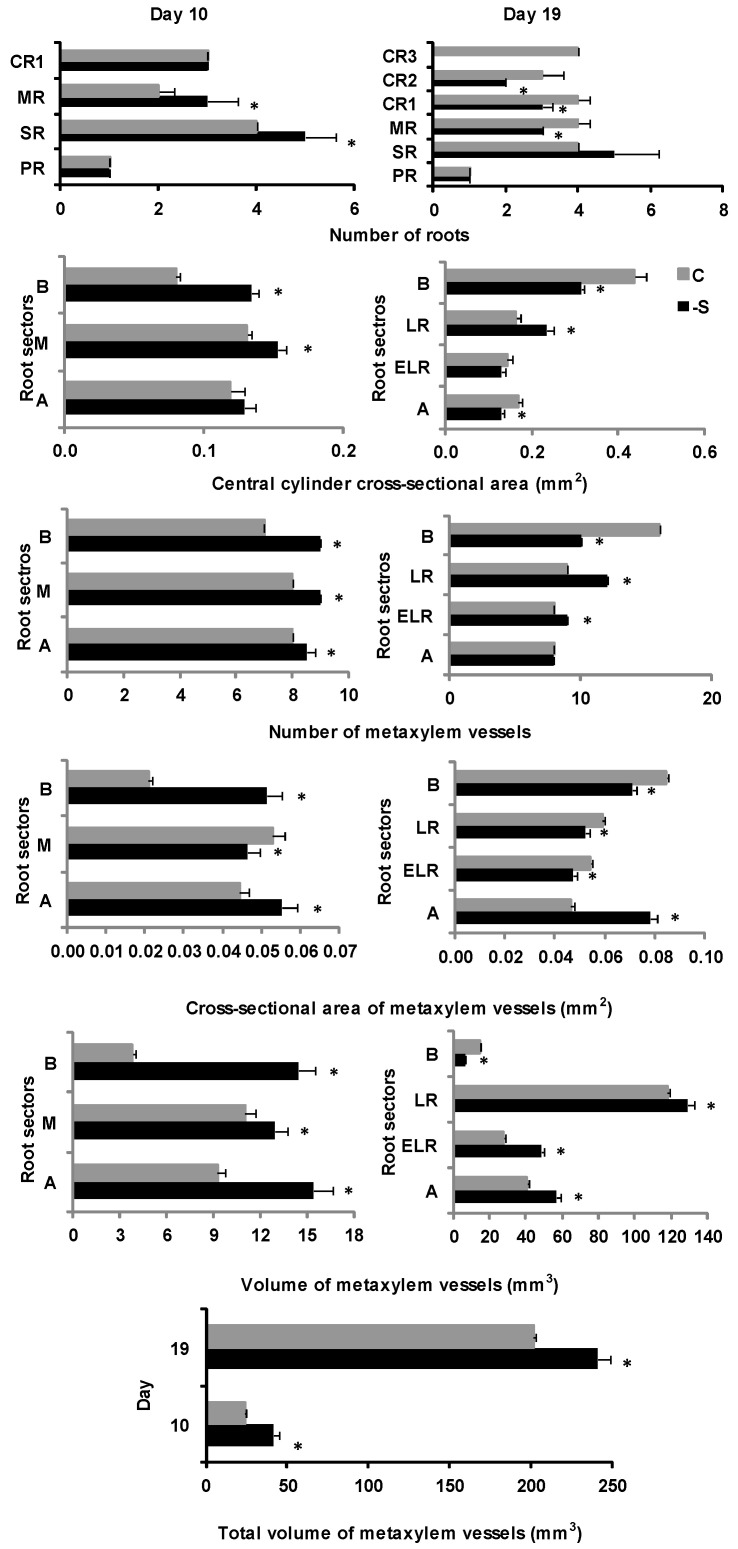
Number of roots, cross-sectional area of the central cylinder, the number, cross-sectional area, volume and total volume of metaxylem vessels (mean ± standard error) at days 10 and 19 of the treatment in plants grown under full nutrition (C, gray columns) vs. sulfate deprivation (-S, black columns). Significant differences (*p* < 0.05) between -S and the respective C are represented by an asterisk (*). PR: primary root, SR: seminal roots, MR: mesocotyl roots, CR1: 1st group of crown roots, CR2: second group of crown roots, CR3: 3rd group of crown roots, B: basal root sector, LR: lateral roots sector, ELR: emerging lateral roots sector, A: root apex, M: middle root sector.

On d19 under S-deprivation, again, all root types had about the same length compared to their control counterparts (Figure S1). The length of the root sector with emerging lateral roots (ELR) of all root types in S-deprived (-S) plants was longer compared to that of control plants (Figure S2). The total number of roots was reduced in comparison to control plants. This reduction was mainly due to a delay in the growth of CR2 and CR3 (Figure 1). The number and length of lateral roots in each root type did not significantly differ compared to control plants, apart from the length of SR lateral roots, which was shorter in S-deprived plants (Figure S1).

#### 2.1.2. Root Anatomical Features Related to the Transport of Nutrients to the Aerial Plant Parts

The root cross-sectional area of maize plants grown for 10 days under S deprivation was greater in all root sectors compared to the control plants. The epidermis and hypodermis cross-sectional area showed an increase from the apical towards the basal sector, and it was larger in -S root sectors than in their control counterparts. The cortex’s cross-sectional area decreased from the apex to the basal sector and was larger in all root sectors than in controls. Aerenchyma had formed in every root sector and increased from the apical sector to the middle region of the root and then decreased near the basal sector. The aerenchymatous area was larger in -S roots than in controls. The central cylinder cross-sectional area appeared to be greater in the middle region of the root compared to the apex and the basal sector and was larger in all root sectors of -S plants compared to controls. The number of metaxylem vessels remained constant in all root sectors of S-deprived plants and was greater than that of control plants. The cross-sectional area of the metaxylem vessels decreased from the apical sector towards the middle region of the root axis and then increased again at the basal sector. Furthermore, the cross-sectional area of the metaxylem vessels was greater in the B and A root sectors of S-deprived roots compared to the B and A sectors of controls. Regarding the volume of the metaxylem vessels, a reduction was observed from the A sector to the middle region of the root and then an increase towards the B sector. However, both the volume of vessels in each root sector and the total volume of vessels were larger than control.

On d19 of sulfate deprivation, the cross-sectional area of S-deprived roots was comparable to that of control roots in general, though the LR root sector under -S presented a larger root cross-sectional area compared to controls (Figure S3). The epidermis and hypodermis cross-sectional area was larger in the LR and ELR root sectors and smaller in the B and A root sectors in the -S treatment compared to the control plants (Figure S3). The LR root sector presented a larger cortex cross-sectional area under S deprivation (Figure S3). Aerenchyma formation was more extensive in the B and A root sectors compared to that observed on d10 and was larger than that of the B and A sectors of control roots (Figure S3).

The central cylinder cross-sectional area was larger in the LR root sector and smaller in the B and A root sectors under S deprivation compared to control conditions (Figure 1). The number of metaxylem vessels in S-starved roots was greater in the LR and ELR root sectors and smaller in the B root sector compared to the control plants (Figure 1). At the same time, the cross-sectional area of the metaxylem vessels was larger in the A sector and smaller in the B sector compared to control conditions (Figure 1). Finally, the volume of the metaxylem vessels appeared to be increased in the LR, ELR, and A root sectors but decreased in the B root sector under -S, thus leading to an increase in the total volume of the metaxylem vessels compared to control (Figure 1).

#### 2.1.3. Plant Foliage Development

**Figure 2:**
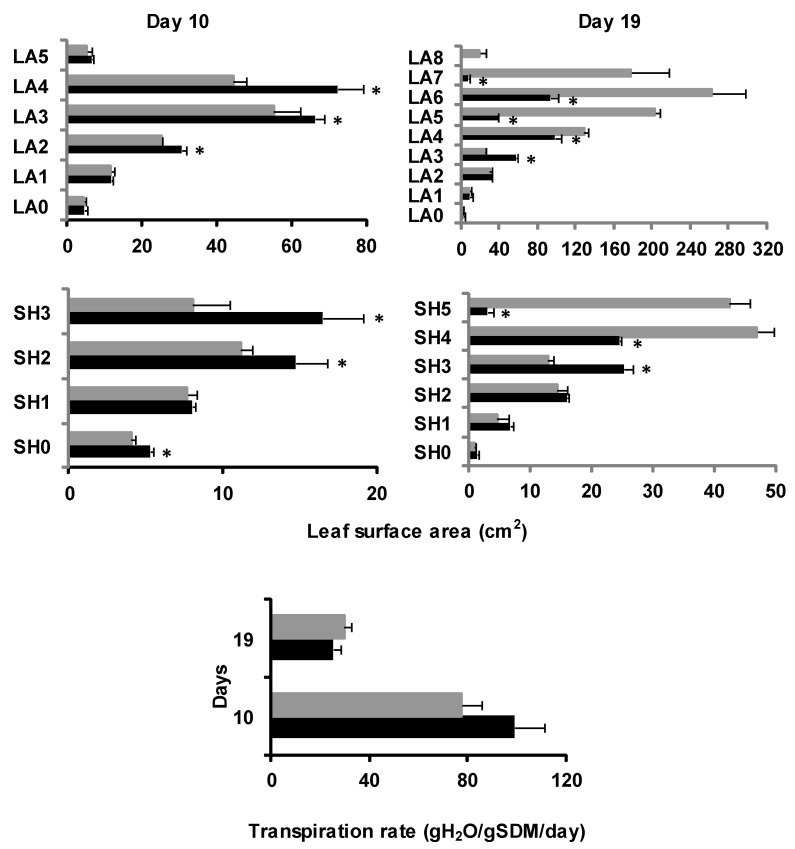
The surface area of each lamina (LA) and sheath (SH) and transpiration rate of the shoot (mean ± standard error) at days 10 and 19 of the treatment in plants grown under full nutrition (C, gray columns) vs. sulfate deprivation (-S, black columns). Significant differences (*p* < 0.05) between -S and the respective C are represented by an asterisk (*). SDM: shoot dry mass.

Under sulfate deprivation on d10, the leaves of S-deprived maize plants were slightly longer as opposed to control plants. On d19, in the shoots of -S plants, the lamina of the sixth leaf (LA6) and the sheath of the fourth leaf (SH4) had developed. The older leaves of S-deprived plants had lengths that did not differ significantly from those of plants grown under full nutrition. However, the younger leaves were shorter under -S when compared to controls (Figure S4). The leaf surface area displayed significant differences between treatments, being greater in the oldest leaves and reduced in the younger ones of S-starved plants compared to controls (Figure 2).

### 2.2. Dry Mass and Water Allocation Dynamics

In the root under S deprivation, on d10, the dry mass presented a similar change pattern, and it was higher in the PR, the SR, and the CR1 compared to controls. The change in pattern followed that of control, and the water content of roots of –S plants was significantly lower than that of control plants.

In the shoot on d10, the allocation among leaves (LAs and SHs) followed that of control plants, and it was either equal in the older leaves or less in the younger leaves compared to the controls.

The change in pattern followed that of the control, and the water content was either higher in the laminas or equal in the sheaths compared to controls.

The transpiration rate of the shoot of the control plants was approximately 77 gH20 gSDM-1 d-1, whilst it was higher in the –S plants by 28%.

In the root on d19, the dry mass of all root types of the -S plants showed an analogous change pattern, and the dry mass of the PR, the MR, the CR1, and the CR2 was greater when compared to control plants (Figure S5). The water content of all root types increased compared to d10 and was either larger or equal to that of controls (Figure S5).

In the shoots of S-deprived plants, dry mass distribution among leaves was similar to that of control plants, and it was either equal in the older leaves or less in the younger leaves compared to the controls (Figure S5). The water content of the younger leaves was higher in S-deprived plants than in controls (Figure S5).

In -S plants, the transpiration rate of the shoot was reduced on d19 compared to d10, and on d19, it was lower under S-deprived conditions than in controls by 16.1% (Figure 2).

### 2.3. Sulfur Allocation Dynamics

On d10 under deprivation, the total sulfur concentration of the roots was slightly increased compared to d0 and in favor of organic sulfur (73%) against sulfates (27%). In each root type, the total sulfur concentration was slightly increased compared to d0 and distributed equally to all root types. -S plants showed a slight increase in sulfate concentration compared to d0, and sulfate concentration was distributed mainly in the PR and SR and less in the MR and CR. The organic sulfur concentration was slightly increased in all root types compared to d0 and distributed almost equally to all root types.

In the shoot, total sulfur concentration was decreased compared to d0 and in favor of organic sulfur (85.1%) against sulfates (14.9%). In each leaf, total sulfur concentration was decreased compared to d0 and distributed about equally to all of the leaves. The sulfate concentration was decreased in the C and L1, increased in the L0 compared to d0, similar to the controls and distributed approximately equal to all of the leaves. It is noteworthy that the sulfate concentration of the L1 was almost equal compared with the controls. The organic sulfur concentration was decreased in the C and L0 and increased in the L1 compared to d0, distributed almost equally to -S leaves, and was lower in all leaves apart from the L0, which was almost equal, compared to the controls.

**Figure 3:**
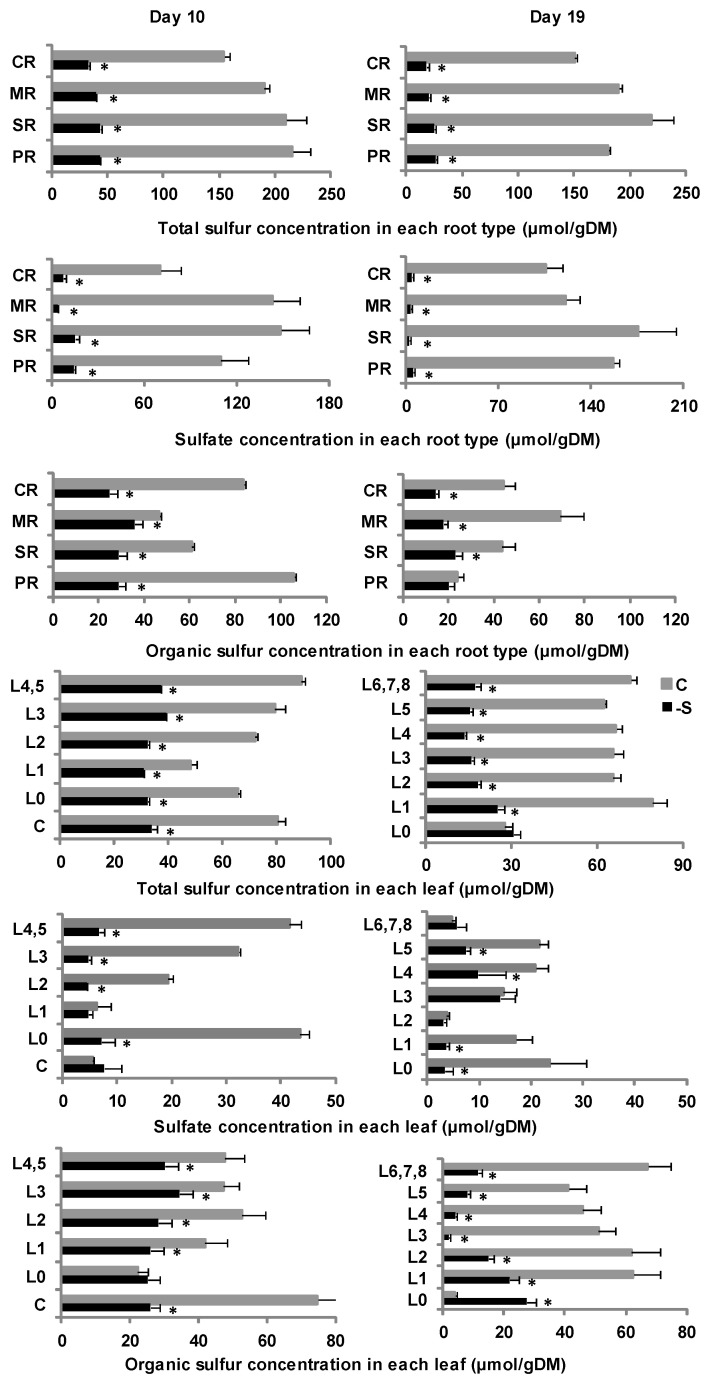
The total sulfur, sulfate and organic sulfur concentration in each root type and in each leaf (L) (mean ± standard error) at days 10 and 19 of the treatment in plants grown under full nutrition (C, gray columns/ gray lines) vs. sulfate deprivation (-S, black columns/ black lines). Significant differences (*p* < 0.05) between -S and the respective C are represented by an asterisk (*). PR: primary root, SR: seminal roots, MR: mesocotyl roots, CR1: 1st group of crown roots, CR2: second group of crown roots, CR3: 3rd group of crown roots, C: coleoptile.

On d19 under deprivation, the total sulfur concentration was diminished compared to d10, reducing sulfates (15%) in favor of organic sulfur (85%) (Figure S6). In each root type, the total sulfur concentration was diminished compared to that on d10 and allocated equally to all root types (Figure 3). The sulfate concentration was reduced in all root types compared to d10 and distributed equally to all root types (Figure 3). The organic sulfur concentration was slightly decreased in all root types compared to d10 and allocated almost equally to all root types (Figure 3).

In the shoot, the total sulfur concentration was diminished compared to d10, reducing organic sulfur (9.6%) in favor of sulfates (90.4%) (Figure S6). The total sulfur concentration was diminished in all leaves compared to that on d10 and was not uniformly allocated with more in the oldest leaves and less in the newer ones (Figure 3). The sulfate concentration of the -S leaves was almost equal to that of the control leaves (Figure 3). The sulfate concentration was decreased in the oldest leaves (L0, L1, L2) and increased in the younger ones (L3, L4) compared to d10 and about equal in the L2, L3, and L6,7,8 compared to the controls (Figure 3). The organic sulfur concentration was only increased in the L0 and decreased in other leaves compared to d10 and was lower in all leaves apart from the L0, which was higher, compared with the controls (Figure 3).

To summarize, in each root type under full nutrition, the total sulfur concentration was composed mainly of sulfates, whilst under sulfate deprivation, it was composed of organic sulfur (Figure S6). In the aerial part under full nutrition, the total sulfur concentration was in favor of organic sulfur against sulfates in all leaves except for the L0 on both days of the treatment. Under S deprivation, on d10, it was in favor of organic sulfur against sulfates in all leaves. On d19, it was in favor of organic sulfur in the L0, L1, L2, L5, and L6, whilst it was composed of sulfates in the L3 and L4 (Figure 3).

### 2.4. Iron Allocation Dynamics

In the root under deprivation, on d10, the total iron concentration was increased compared to d0 and in favor of internal iron (95.2%) against apoplastic iron (4.8%). The total iron concentration in each root type was increased compared to d0 following a different pattern, which was PR > SR > CR > MR and it was less than control. The apoplastic iron concentration was increased in the PR and slightly decreased in the SR compared to d0, following a similar change pattern as the control. It was less in the PR, SR, and CR and more in the MR than the control. The internal iron concentration was more in all root types than d0 and presented the same change pattern as that of total iron on the corresponding day and treatment. It was also less in all root types compared with control.

As for the contribution of internal iron and apoplastic iron in each root type, under sulfate deprivation, on d10, the internal iron was more in the PR, the SR, and the CR and less in the MR.

On d10, the total iron concentration of the shoot was decreased compared to d0 and was less than control plants. The total iron concentration was increased in the L0 and decreased in the L1 compared to d0, following a different pattern since it decreased from the lower to the upper leaves and was higher in the L0, equal to L1, and lower in the other leaves than control plants.

**Figure 4:**
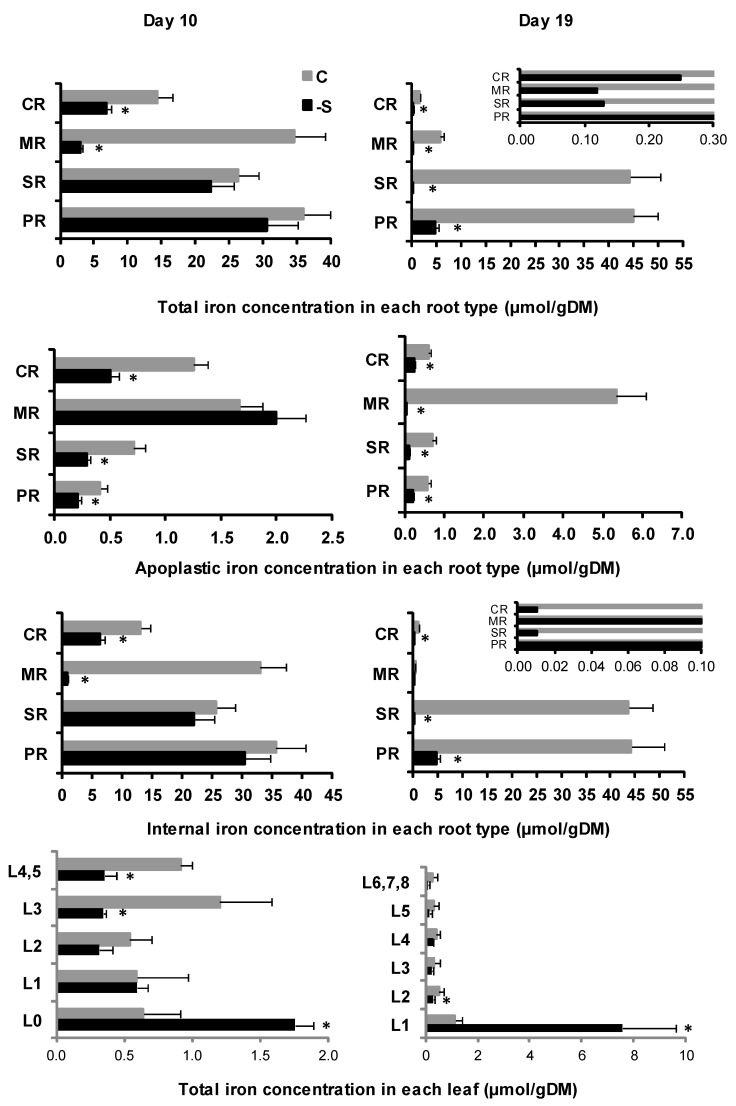
The total, internal and apoplastic iron concentration in each root type and the total iron concentration in each leaf (L) (mean ± standard error) at days 10 and 19 of the treatment in plants grown under full nutrition (C, gray columns/ gray lines) vs. sulfate deprivation (-S, black columns/ black lines). Significant differences (*p* < 0.05) between -S and the respective C are represented by an asterisk (*). PR: primary root, SR: seminal roots, MR: mesocotyl roots, CR: crown roots.

In the root under sulfate deprivation, on d19, the total iron concentration was dramatically diminished compared to d10 and in favor of internal (89.3%) vs. apoplastic iron (10.7%) (Figure S6). The total iron concentration was drastically decreased in all root types compared to d10 and was less compared with control plants (Figure 4).

The apoplastic iron concentration was less in the SR, the MR, and the CR and remained the same in the PR compared to d10 and less in all root types compared to the control (Figure 4).

The internal iron concentration was reduced dramatically in all root types compared to d10 and was less in all root types compared to the control (Figure 4).

Under sulfate deprivation on d19, the internal iron was more than apoplastic iron in the PR and MR and less in the SR and CR.

In the shoot under the deprivation, the iron allocation was raised compared to d10 and was more than the control (Figure S6). Total iron concentration in each leaf presented a similar change pattern compared to control leaves and was much higher in L1 than control (Figure 4).

## 3. Discussion

### 3.1. Root Morphology—Traits Involved in Exploration and Nutrient Uptake

It is well known that under nutrient deficiency, plants actuate foraging responses that contain root morphological changes, such as the modification of root system architecture or root hair development [34,35,36,37].

In the present study, plants grown under sulfate deprivation did not seem to invest in new root axes but to try to optimize the nutrient uptake efficiency of existing roots. The fewer root axes were due to the fact that each group of crown roots showed one less root axis, and CR3 did not appear, and only the SR increased the number of their axes. As a result, the deprivation changed the number of axes in each root type and decreased the overall number of axes. According to Song et al. [38], *Pistacia chinensis* plantlets decreased the negative consequences of a lack of nutrients by fostering root growth and developing N and K distribution in storage organs. In Gao et al. [39], the number of the seminal roots and the first group of crown roots of maize plants grown under N deficiency was not considerably affected, demonstrating that root onset was not affected soon by N deficiency, whilst with extended low nitrogen stress, the growth of CR1 was inhibited, and the numbers of CR2 and CR3 were greatly reduced. It was also demonstrated that fewer axile roots under low nitrogen stress are produced in maize [40,41].

In contrast to the number of root axes, -S plants seem to form the root system architecture to cope with sulfate deprivation (Table 1). Comparing -S with C plants, both preserved about the same length of each root type throughout the experiment. This finding is consistent with a previous result that sulfur deficiency had little effect on primary root elongation in Arabidopsis [42]. Nevertheless, -S plants increased the length of the root sector carrying lateral roots in the PR and MR on d10 and the length of the root sector with emerging lateral roots in all root types on d19. Furthermore, during d10 of the treatment, both PR and SR had more and shorter lateral roots compared to control plants. Thereafter, the root system of S-deficient plants was enhanced at various levels to explore a wider area of the substrate towards better access to the nutrient solution. In solution culture, Wang et al. [40] and Tian et al. [43] noticed that low nitrogen stress decreased the total length of lateral roots in maize plants. Furthermore, under S-deficient conditions, root growth in Arabidopsis was improved, resulting in more lateral roots and greater root hair density [44]. In addition, P-deficient conditions induce lateral root initiation and emergence [45]. On the contrary, the study of lateral root-defective rice (*Oryza sativa*) mutant showed that lateral roots contribute considerably to the acquirement of phosphorus, manganese, zinc, and copper, while their contribution was less meaningful for the mobile nutrients nitrogen and sulfur [46]. Moreover, Postma et al. [47] concluded that maize growth under low nitrogen or phosphorus accessibility is susceptible to lateral root branching density. Greater and shorter lateral roots is an advantageous trait for phosphorus acquisition, whilst fewer and longer lateral roots is an advantageous trait for nitrate acquisition.

### 3.2. Root Anatomy—Traits Involved in Transport/Translocation of Nutrients

S-deficient plants appear to invest in the development of the radial growth of the crown root in all sections on d10 and in some of them on d19 (Table 1). Specifically, CR1 presented anatomical differences in all parameters studied on d10, i.e., it had larger root cross-sectional area, epidermis and hypodermis, cortex, aerenchymatous area, central cylinder, number and cross-sectional area of xylem vessels, the volume of vessels in each root sector, as well the total volume of vessels. On d19, it exhibited differentiations, one of them was that the aforementioned parameters were larger in the root sector carrying lateral roots (LR) and smaller in the basal root sector (B) compared to the control plants.

The larger number, cross-sectional area, and volume of the xylem vessels indicate that -S plants have created a better-equipped vascular system for more effective transport of nutrients from the root system to the above-ground part of the plant. More and larger xylem vessels serve the increased transport of water and nutrients in the xylem sap. Our data also confirmed that under sulfate deprivation, the aerenchymatous area was larger on both days of treatment. This might happen to support the lateral roots by inducing cell death and recycling the liberated material [48].

To date, aerenchyma formation has been reported in maize crown roots by lysis of cortical cells under nutrient shortage, especially nitrogen, phosphorous, and sulfur [49,50,51,52].

According to Lynch [53], anatomical features of a “steep, deep and cheap” root system ideotype contain attributes that decrease the metabolic cost of soil exploration, for instance, the configuration of root cortical aerenchyma (RCA), decreased cortical cell number, increased cortical cell size, and root cortical senescence and attributes that increase root penetration of hard subsoils. Genotypic alteration for RCA formation is associated with enhanced N acquisition in maize [54]. Simulation modeling demonstrates that root cortical senescence enhances N acquisition in barley [55] and that reduced cortical cell number and increased cortical cell size enhance N acquisition in maize [56]. Root anatomy adjusts the penetration of hard soils and is correlated to rooting depth in maize [57], which ought to increase N capture [58]. RCA diminishes the respiration and P cost of retaining root tissue and thus enhances P capture by maize and bean in silico [59,60]. RCA also benefits K capture [59,60] and root cortical senescence [55].

### 3.3. Root Nutrients

The reduced concentration of sulfur was observed in the roots of -S plants. Further, organic sulfur predominated over sulfates throughout the experiment. Despite the S-deprivation treatment, the roots of -S plants continued to accumulate sulfur in small quantities during the experiment. As elucidated in previous work, the existing decreased sulfur emanated from the seed stocks, as well as the impurities given by each reagent used to make the nutrient solution [61].

In this work, total sulfur concentration increased on d10 under the deprivation and decreased on d19 in all root types, containing more organic sulfur than sulfates on both days of the treatment. Under full nutrition, the total sulfur concentration increased in all root types on d10 and decreased in three out of the four root types (it increased only in the SR) on d19, containing more sulfates than organic sulfur on both days of the treatment.

This result is supported by Astolfi et al. [62], who analyzed the interplay between S and Fe nutrition in the root system. It has been demonstrated that both enzymes ATP sulfurylase and O-Acetylserine sulfydrylase involved in S metabolic pathway were stimulated by S deficiency to create and preserve adequate cellular pools of reduced sulfur for cellular functions. On the contrary, under full nutrition, roots do not appear to contribute significantly to the plants’ need for reduced sulfur. Reduced sulfur compounds are missing from xylem sap or are present solely in low concentrations [63].

In the root system of S-deficient plants, a reduced concentration of total iron was observed. This reduction was noticed on d19 of sulfate deprivation. As regards the distribution of total iron in each root type, our data revealed that under the deprivation, there were differences in the subcellular localization of iron (Fe_INT_ and Fe_APO_). The percentage distribution of Fe_APO_ in each root type is given in Table 2. Specifically, in the PR, the majority of iron was internal iron throughout the experiment in both treatments. Under sulfate deprivation, in the SR and CR, most of the iron was internal iron on d10 and apoplastic iron with a percentage over 95% on d19. In contrast, in the MR, the largest amount of iron is apoplastic on d10 and internal on d19. Furthermore, under deprivation, the PR, SR, and CR followed the same change pattern, and the percentage of apoplastic iron was higher than that of control plants.

It has been plainly shown that restricted S availability prevents the plant’s ability to uptake and accumulate Fe by diminishing the rate of PS release in grasses [64,65,66,67]. Sulfur-deficient conditions cause a considerable increase in the requirement for S and, therefore, activates S uptake and assimilation rate. In particular, S deficiency considerably increased the rates of ^35^SO_4_^2–^ uptake from maize and barley roots [62,68]. In addition, S deficiency influenced the distribution of the reduced S pool from the shoots to the roots within the plant: -S barley plants presented a raised-root cysteine concentration by enhanced ATPS activity and translocation from the shoot [68]. Sulfur sufficiency regulates the expression level of genes associated with both uptake and assimilation of sulfate in grasses, for example. barley and durum wheat [30,31,68].

In the present study, the largest amount of iron under full nutrition is internal iron in all root types throughout the experiment, except MR, where it is apoplastic iron on d19. In similar studies with plants grown in nutrient solution cultures, the root apoplast has been hypothesized to be significantly enriched in Fe [69,70]. Moreover, in soil-grown plants, the apoplasmic Fe pool, laden with miscellaneous indigenous Fe compounds, can be a substantial Fe source in graminaceous species and a source of Fe removing [71]. Masalha et al. [72] have presented that nearly 50% of the iron accumulated in maize roots grown in the soil was released through chemical reduction with sodium dithionite. Kosegarten and Koyro [73] have considered that almost 65% of the iron concentration in maize roots was released during chemical reduction. Other investigations have assumed that the apoplastic Fe was overrated in plant roots growing in the soil as a result of Fe soil contamination at the root surface and the apoplastic Fe represent less than 40% of the total root Fe [74]. Our study confirmed that apoplastic iron in each root type, after removing iron precipitations from the root surface with DCB treatment, was less than internal iron; apoplastic iron percentage varied among root types under the deprivation. In three out of the four root types (PR, SR, CR), the internal iron was more on d10, and in two of the four (PR and MR), on d19. Additionally, our data revealed that the apoplastic iron percentage was more in the MR on d10 and in the PR, SR, and CR on d19 compared to control plants. Hence, in cases where apoplastic iron is higher in -S plants than in control ones, it may be explained by considering that maize as a graminaceous plant utilizes Strategy II to acquire Fe and that methionine is necessary for the PSs biosynthetic pathway [75]. The PSs can form stable complexes with iron, and the entire complex of Fe–PS is transferred via the cell membrane. Thus, S starvation could lead to decreased methionine and PSs production and apparently affects Fe mobilization from the apoplastic space.

### 3.4. Aerial Plant Parts and Nutrients’ Translocation

In this work, the aim was also the detailed morphometric analysis of the foliage and the determination of the transpiration rate. Our results revealed that under S deprivation, on d10, the dry mass was either equal in the older leaves or less in the younger ones, both the water content and the leaf length were almost equal, the surface area was longer, and the transpiration rate of the shoot was higher compared to control (Table 1). On d19, the dry mass was either equal in the older leaves or less in the younger ones, the water content of the functional leaves was higher, the leaf length was similar in older leaves or shorter in the younger ones, the surface area, in some cases, was greater, and the transpiration rate of S-deprived plants was lower compared to control on the same day (Table 1).

Taken together, S-deficient plants do not differ in terms of dry mass and leaf length (laminas and sheaths), while they predominate in terms of the surface area of the laminas and sheaths compared to control plants during the first ten days. The dry mass, length, and surface area of the upper leaves (laminas and sheaths) are most affected by the lack of sulfates during the second ten days.

In terms of the transpiration rate, comparing -S with control plants, on d10: (i) the dry mass of the aerial part was lower by 20.6%, (ii) the dry mass of the root system was higher by 40%, (iii) the surface area was higher, and (iv) the characteristics of the vascular system of the roots were appropriately modified by increasing the cross-sectional area of xylem vessels, apparently to increase the water flow and nutrients to the leaves. The immediate consequence of these changes was an increase in the transpiration rate by 28.2%. On the other hand, on d19 in S-deficient plants: (i) the dry mass of the aerial part was significantly lower by 62%, (ii) both the dry mass of the root system and the surface area did not differentiate, and (iii) the cross-sectional area of xylem vessels decreased compared to control plants. These results might justify a 16.1% reduction in the transpiration rate of the above-ground plant part.

As expected, the S deprivation negatively affected the total sulfur concentration in the aerial plant part. It was allocated as organic sulfur by 85.1% on d10 and sulfates by 90.4% on d19.

It is noteworthy that the sulfate concentration in leaves was the same in both control and -S plants, while the organic sulfur concentration was much higher in control plants than -S ones ond19, and the sulfate concentration of the leaves in -S plants was much higher than the organic sulfur concentration on the same day (Table 1).

In the shoot of S-deficient plants, the total sulfur concentration decreased in all leaves on both days of the treatment. It was noticed that the sulfate concentration in specific leaves was almost equal compared to the controls as, for instance, the L1 on d10, or L2, L3, and L6,7,8 on d19. Additionally, the organic sulfur concentration was lower in all leaves compared to control plants, except for the L0 that was approximately the same on d10 and much higher on d19.

To summarize, under sulfur deficiency, plants accumulate sulfur in small quantities, mainly in the form of sulfates in the aerial part and organic sulfur in the roots. This conclusion is consistent with previous studies. Astolfi et al. [76] showed that sulfate deprivation determined a reduction in the level of non-protein SH compounds in maize leaves compared to control ones. Moreover, in their experimental conditions, ATP sulfurylase and O-Acetylserine sulfydrylase activities were oppositely related to the leaf sulfate content. Actually, maize leaves showed an increase both of ATP sulfurylase and O-Acetylserine sulfydrylase activities after 10 days of sulfate deprivation. Their results justify the increased percentage of organic sulfur observed in our results on the same day.

However, sulfate accumulation in maize leaves under sulfur-deficiency stress could be explained by the fact that a unique regulatory mechanism performs through the formation of an enzyme complex comprising Ser acetyltransferase and OAS (thiol)-lyase, two enzymes committed to the terminal step of sulfur assimilation. Complex stabilization is inversely controlled by OAS and sulfide. OAS accumulation stimulated by sulfur deficiency promotes dissociation of the complex to moderate Ser acetyltransferase activity, resulting in reduced OAS formation. In succession, with increased sulfur supply, accumulated sulfide promotes the formation of the complex, leading to stimulated OAS formation to accomplish the Cys synthesis. This system permits the coordination of OAS synthesis from Ser and sulfate reduction for efficient Cys production [77]. It is widely known that the activity of sulfate uptake and assimilation is caused by sulfur deficiency stress or a high need for sulfur metabolites. Sulfur starvation leads to an increase in OAS levels, which sequentially causes the genes’ expression encoding sulfate transporters and APS reductase, thus superseding the restrictive effect of sulfur-sufficient nutritional conditions [78,79]. In contrast to OAS’s positive effect, thiols, such as Cys and GSH, operate as negative regulators of sulfur metabolism.

Regarding the iron concentration in the aerial part, our data revealed that under sulfate deprivation, it decreased on d10 and slightly increased on d19. On the other hand, in the roots of S-deficient plants, it was dramatically decreased on d19. Consequently, this reduction may be due to the movement of the iron to the leaves. The results of the iron concentration in each leaf indicated that -S plants accumulated excessive iron in the lower leaves, such as in the L0 on d10 and the L1 on d19. In summary, the effect of sulfur deficiency led to a different iron allocation in the foliage and a significant reduction of iron in the upper (younger) leaves of the aerial part of -S plants.

It is known that under sulfur deficiency, maize plants had a reduced shoot Fe content than those grown under complete nutrition [64,76,80]. Moreover, it has been shown in barley that S deficiency could probably prevent iron accumulation in shoots by decreasing the rate of PS release [65] and/or by restricting the ability to take up iron from the external solution [68]. Additionally, HvYS1 expression, the particular transporter of Fe^3+^-PS complexes, is adjusted by S supply [29] in barley, indicating that S mostly affects the Fe uptake step.

Apart from this fact, it has been lately shown that the reduction of iron content in rice shoots due to S-deficiency was associated with a decreased nicotianamine (NA) level, implying that S is not only important for iron uptake but also for its mobilization to the shoot [81], with NA being the major iron-chelating agent included in both xylem and phloem Fe transportation in plants [25,82].

## 4. Materials and Methods

### 4.1. Plant Material and Hydroponics Set Up

Maize seeds (Zea mays “Cisko”, Syngenta Hellas) were preserved on wet filter paper in the dark at 28 °C and relative humidity of 76% until germination. After four days, the most uniform of those plants were chosen and retained in hydroponic batch culture for 3 days in well-aerated, distilled H_2_O. A regulated environment of 250 μmol photons m^−2^ s^−1^ photosynthetic photon flux density (PPFD) and a 14h light photoperiod with day/night growth conditions at shoot base 28/23 °C and RH 36/40% was used.

### 4.2. Treatments and Samplings

On the seventh day after sowing and for the next 19 days, hydroponic batch cultures were performed using two nutrient solutions. Half of the plants were grown in complete nutrient solution (C), while the rest were grown in S-deprived nutrient solution (-S). The complete nutrient solution contained 5 mM KNO_3_, 1 mM KH_2_PO_4_, 2 mM Mg(NO_3_)_2_, 2.5 mM CaSO_4_, 1 mM MgSO_4_, 0.07 mM EDTAFeNa, 4 mM Ca(NO_3_)_2_, 0.9 μM ZnCl_2_, 30 μM H_3_BO_3_, 0.9 μM CuCl_2_, 0.5 μM MoO_3_, and 20 μM MnCl_2_. The S-deprived nutrient solution contained 5 mM KNO_3_, 1 mM KH_2_PO_4_, 2 mM Mg(NO_3_)_2_, 0.07 mM EDTAFeNa, 4 mM Ca(NO_3_)_2_, 0.86 mM CaCl_2_, 0.9 μM ZnCl_2_, 30 μM H_3_BO_3_, 0.9 μM CuCl_2_, 0.5 μM MoO_3_, and 20 μM MnCl_2_. All nutrient solutions were continually aerated and replaced every 3 days. The samplings were performed at day 0 prior to separation into two nutrient solutions, at day 10, and 19 of their separation.

### 4.3. Morphometric Analysis

The maize root system contains one primary (PR) and a few seminal roots (SR), which constitute the embryonic root system and the crown roots, i.e., the consecutive nodal roots (CR1, CR2, CR3), which together with the lateral roots constitute the post-embryonic system [83]. In addition, the cultivated variety utilized in this research creates mesocotyl roots (MR).

Each root type was divided into four sectors, the basal root sector (B), the root sector carrying lateral roots (LR), the root sector with emerging lateral roots (ELR), and the apical root sector (A) [48]. The morphometry of each root type was reported concerning root length and its root sectors, the number of roots of each root type, and the number and length of the lateral roots. In addition to the root system, the parameters measured were the length of each lamina (LA) and sheath (SH) and the corresponding leaf area on the three sampling days. For this purpose, the phytomers were placed on a calibrated paper and photographs were taken and analyzed by using the Image J software (https://imagej.nih.gov/ij/, accessed on 12 April 2020).

### 4.4. Histological Study

The scope of this study was to examine the anatomy of maize roots grown under sulfate deprivation. Due to the complexity of the architecture of the maize root system, our study focused on the 1st group of crown roots (CR1) because these roots are the first to appear after the embryonic root system, as well as crown roots that constitute the main root system of the maize plant. To achieve this goal, samples from each root sector were taken using the method described thoroughly in an earlier study [61]. A number of anatomical parameters were determined, i.e., root cross-sectional area, epidermis and hypodermis, cortical cells, central cylinder cross-sectional area, aerenchymatous area, along with the number and cross-sectional area of metaxylem vessels. The volume of metaxylem vessels in each root sector was calculated by multiplying the number of metaxylem vessels in each root sector by its cross-sectional area and its length. The total volume of metaxylem vessels was then calculated by adding the volume of metaxylem vessels of all root sectors.

### 4.5. Dry Mass Determination

Fresh weight per phytomer was calculated, plant parts were oven-dried at 80 °C, and dry weight was reported. Synthesized samples of the suitable dry mass were then ground to pass through a 40 mesh sieve utilizing an analytical mill (IKA, model A10) prior to chemical analysis [84].

### 4.6. Transpiration Rate Determination

The transpiration rate was determined as described in Maniou et al. [61]. At d9 and d18 of the treatments, four vessels of 1 L each, covered with aluminum foil, were used. A nutrient solution was added to each vessel to a final weight of 1000 g, thus: complete nutrient solution in the first vessel and complete nutrient solution with 1 plant in the second one, S-deprived nutrient solution in the third vessel, and S-deprived nutrient solution with 1 plant in the fourth one. After 24 h, the plants were removed, the vessels were weighed, and the mass of water lost was recorded. Three repetitions of each determination were performed.

### 4.7. Sulfate Determination

The sulfate concentration (SO_4_) was identified by extracting of the oven-dried samples with 2% (*v*/*v*) water solution of the acetic acid and by determining with the turbidimetric method [85,86].

### 4.8. Total Sulfur Determination

The total sulfur concentration (S_TOT_) was identified after dry ashing at 600 °C [76]. The ash was diluted in a 2% (*v*/*v*) water-based solution of the acetic acid, infiltrated with Whatman No.42 paper, and total sulfur concentration was identified with the turbidimetric method [85,86].

### 4.9. Organic Sulfur Calculation

The organic sulfur (S_ORG_) per phytomer and per day was estimated by removing the sulfate (SO_4_) amount from the total sulfur (S_TOT_) amount.

### 4.10. Total Iron Determination

The oven-dried samples were digested with warm H_2_SO_4_ and successive additions of 30% H_2_O_2_ until complete digestion, and then the total Fe (Fe_TOT_) in the diluted digestion products was measured by atomic absorption spectrophotometry (GBC, Model Avanta spectrophotometer) [84].

### 4.11. Apoplastic Iron Determination

Apoplastic iron (Fe_APO_) stands for all iron segments found within the area defined by the plasma membranes and the plant surface. Iron depositions attached to the root surface are not contained. With the aim of taking away any iron depositions from the root surface, the usage of dithionite-citrate-bicarbonate (DCB) was applied [87,88,89]. According to this method, at harvest, the entire root system of each plant was incubated for 60 min at room temperature (20–25 °C) in 40 mL of a solution consisting of 0.03 M sodium citrate (Na_3_C_6_H_5_O_7_.2H_2_O) and 0.125 M sodium bicarbonate (NaHCO_3_), with the addition of 0.6 g sodium dithionite (Na_2_S_2_O_4_). Na_2_S_2_O_4_ is a potent reducing agent in NaHCO_3_ solution that reduces ferric iron; the ferrous iron is complexed and transferred with citrate. The roots were washed three times with deionized water, and then the Bienfait method was implemented to estimate the apoplastic iron in each root type [69]. According to this method, the dithionite reagent reduces the ferric iron, and the ferrous iron reacts with bipyridyl to generate a purple color. The release of K^+^ throughout this reductive mobilization of iron from the roots was utilized as an index of tissue injury, and thus, the extraction procedure was adjusted to 7 min.

### 4.12. Internal Iron Calculation

Internal iron (Fe_INT_) is the sum of symplastic iron and non-extractable iron located in apoplastic space (including the iron found in the xylem vessels). Internal iron per phytomer and per day was estimated by removing the apoplastic iron (Fe_APO_) quantity from the total iron (Fe_TOT_) quantity.

### 4.13. Statistical Analysis

Every treatment (C, -S) was reiterated three times with the implementation of three distinctive hydroponic experiments. In each reiteration, a number of plants were obtained, which provided a sufficient amount of dry mass, and the composite sample was utilized for chemical analyses; three composite samples were individually examined. The comparisons between the corresponding values of -S and C were subjected to a *t*-test analysis of variance with a two-tailed distribution and two-sample equal variance, at *p* ≤ 5%. Where the differences between the means of samples C and -S were statistically important, the percentage of the relative change is indicated by an asterisk. The software used for statistical analysis was Microsoft Excel.

## 5. Conclusions

In young maize plants, phenotypic adaptation to sulfur deprivation in maize over time presented alterations mainly at the anatomical level, in all parameters studied, coupled with a dynamic profile. The phenotypic adaptation is coupled with sulfur and iron allocation in the root system and the aerial part. The main findings of this study were that when maize plants are grown under sulfate deprivation:

- They did not invest in new root axes.
- They developed more and larger xylem vessels in roots.
- Organic sulfur predominated over sulfates into their roots.
- Sulfates predominated over organic sulfur into their leaves.
- Τhere were differences in the subcellular localization of iron in their roots.

Those adaptations resemble an attempt to maximize water and nutrient long-distance transport to the shoot, towards competent handling of the initial sulfur and the induced iron deficiencies.

## Figures and Tables

**Table 1:** Towards the broad picture: combining phenological and nutritional dynamics. x: organ or tissue not existed. =: same value between C and -S. C: value of C > -S. -S: value of -S > C.

Leaf 5 *					Day 10		Day 19			
Leaf length LA5					=		C			
Leaf surface area LA5					=		C			
Dry mass LA5					C		C			
Water content LA5					-S		-S			
Total sulfur concentration					C		C			
Sulfate concentration					C		C			
Organic sulfur concentration					C		C			
Total iron concentration					C		C			
* the youngest lamina at day 10										
Leaf 3 **					Day 10		Day 19			
Leaf length LA3					=		=			
Leaf surface area LA3					=		-S			
Dry mass LA3					=		=			
Water content LA3					=		-S			
Total sulfur concentration					C		C			
Sulfate concentration					C		=			
Organic sulfur concentration					C		C			
Total iron concentration					C		C			
** constant lamina length between days 10 and 19										
(its development has been completed)										
Shoot					Day 10		Day 19			
Transpiration rate					-S		=			
Total sulfur concentration					C		C			
Sulfate concentration					C		=			
Organic sulfur concentration					C		C			
Total iron concentration					C		(-S)			
	C	C	C	CR1		Day 10	Day 19	C	C	C
	C	C	C	Length of sector	B	x	=	C	C	C
	Total sulfur concentration	Sulfate concentration	Organic sulfur concentration		LR	x	(C)	Total iron concentration	Apoplastic iron concentration	Internal iron concentration
					ELR	x	-S			
					A	=	(C)			
				Root cross sectional area	B	-S	C			
					LR	-S	-S			
					ELR	-S	=			
					A	-S	C			
				Epidermis and hypodermis cross-sectional area	B	-S	C			
					LR	-S	=			
					ELR	-S	-S			
					A	-S	C			
				Cortical parenchyma cross-sectional area	B	-S	C			
					LR	-S	-S			
					ELR	-S	C			
					A	-S	C			
				Aerenchymatous area	B	-S	-S			
					LR	-S	-S			
					ELR	-S	C			
					A	-S	-S			
=	C	(C)	=	Central cylinder cross-sectional area	B	-S	C			
=	=	x	x		LR	-S	-S			
					ELR	-S	=			
Root length	Number of roots	Number of lateral roots	Length of lateral roots		A	=	C			
				Number of metaxylem vessels	B	-S	C	=	(-S)	
					LR	-S	-S			
					ELR	-S	-S	-S	C	
					A	-S	=			
				Cross-sectional area of metaxylem vessels	B	-S	C	Dry mass	Water content	
					LR	C	C			
					ELR	C	C			
					A	-S	-S			
				Total volume of metaxylem vessels		-S	-S			

**Table 2:** The percentage distribution of Fe_APO_ fractions in each root type under complete nutrition against sulfate deprivation.

	Day	PR	SR	MR	CR
		% Fe_APO_			
**C**	0	43	78		
	10	1	3	5	9
	19	1	2	94	38
**-S**	0	43	78		
	10	1	1	67	7
	19	4	95	14	97

## Data Availability

The data presented in this study are available on request from the corresponding authors. The data are not publicly available due to privacy.

## Supplementary Materials

The following supporting information can be downloaded at: https://www.mdpi.com/article/10.3390/plants11050703/s1, Figure S1: Root length, number and length of lateral roots of each root type; Figure S2: The length of the sectors in each root type and the percentage it occupies on the total length of the plants’ roots; Figure S3: Cross-sectional area of the root, epidermis and hypodermis, cortical parenchyma and aerenchymatous area; Figure S4: Length of each leaf’s lamina and sheath; Figure S5: The allocation of the dry mass and water content in each root type and in each leaf’s lamina and sheath; Figure S6: The total sulfur, sulfate and organic sulfur concentration in the root system and in the aerial plant part.
