# Supplementary material for: Phenotypic Acclimation of Maize Plants Grown under S Deprivation and Implications to Sulfur and Iron Allocation Dynamics
Source: Plants (Basel). 2022 Mar 6;11(5):703. doi: 10.3390/plants11050703 (PMC8912738; doi:10.3390/plants11050703)
Supplement: Supplementary file 1 [file plants-11-00703-s001.zip › plants-1614217-supplementary.pdf]

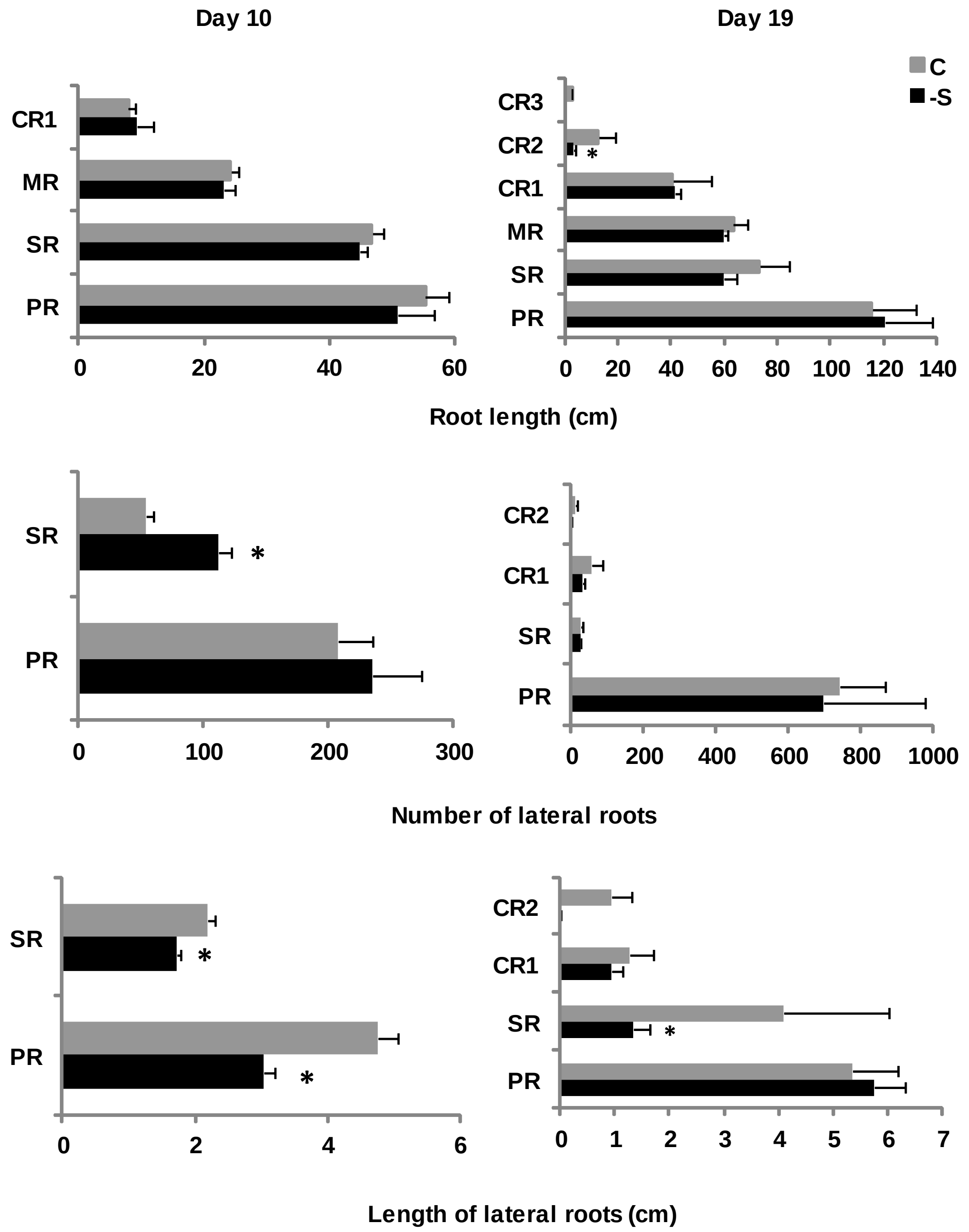

Figure S1. Root length, number and length of lateral roots of each root type (mean ± standard error) at days 10 and 19 of the treatment in plants grown under full nutrition (C, gray columns) vs sulfate deprivation (-S, black columns). Significant differences ($p < 0.05$) between -S and the respective C are represented by an asterisk (*). PR: primary root, SR: seminal roots, MR: mesocotyl roots, CR1: 1st group of crown roots, CR2: second group of crown roots, CR3: 3rd group of crown roots.

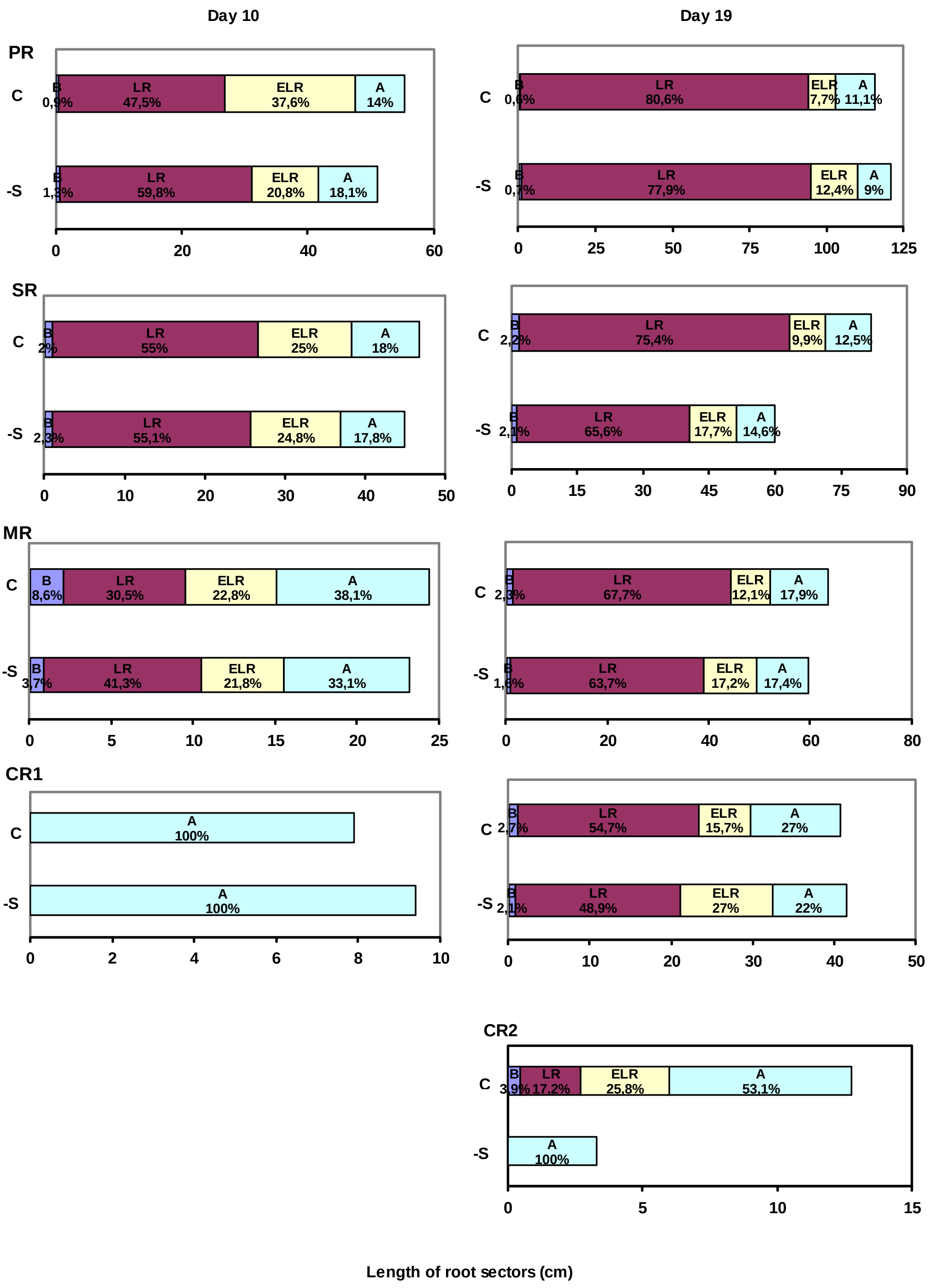

Figure S2. The length of the sectors in each root type and the percentage it occupies on the total length of the primary root (PR), the seminal roots (SR), the mesocotyl roots (MR), the 1st and 2nd group of crown roots (CR1, CR2) at days 10 and 19 of the treatment in plants grown under full nutrition (C) vs sulfate deprivation (-S). B: basal root sector, LR: lateral roots sector, ELR: emerging lateral roots sector, A: root apex.

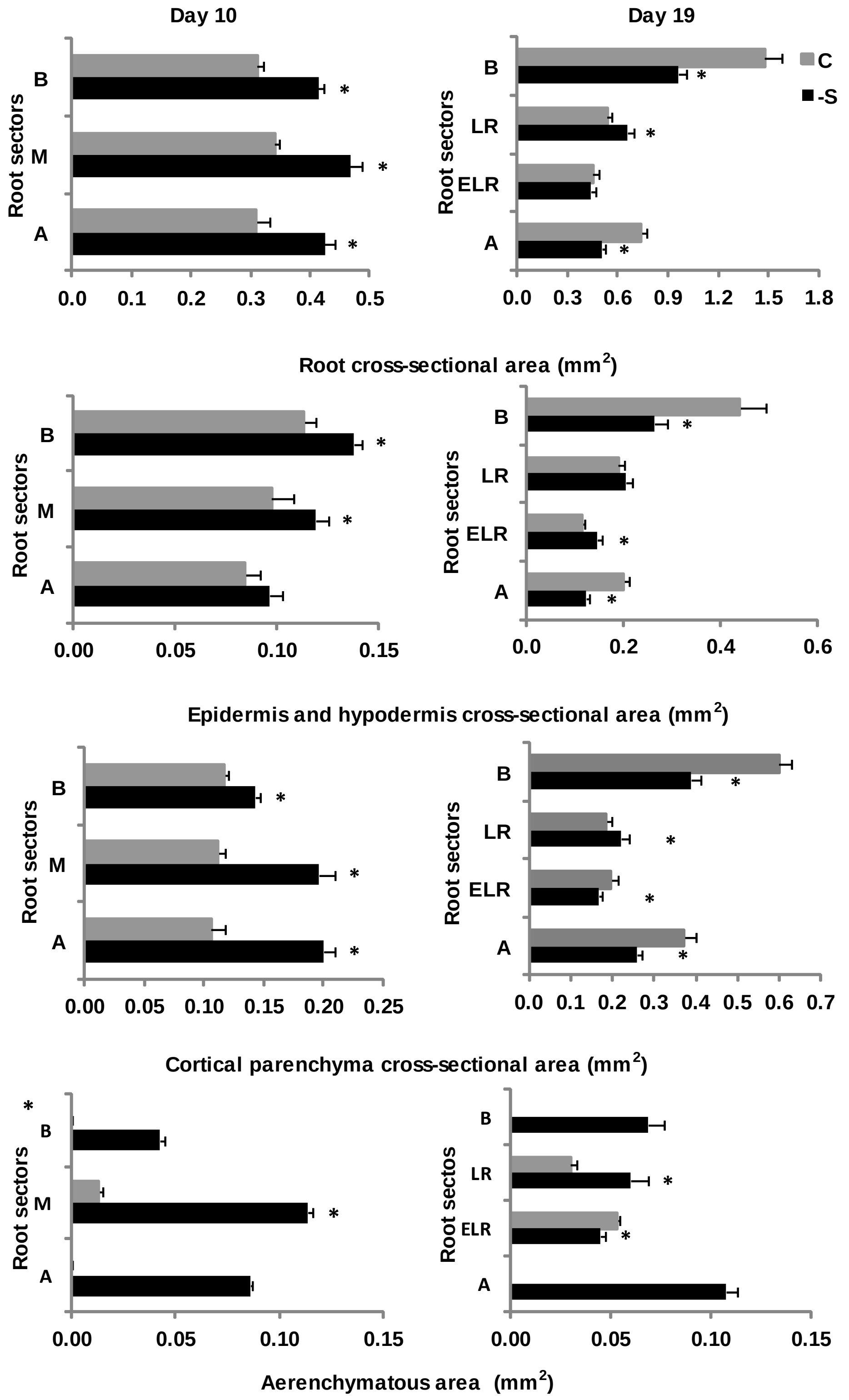

Figure S3. Cross-sectional area of the root, epidermis and hypodermis, cortical parenchyma and aerenchymatous area (mean ± standard error) at days 10 and 19 of the treatment in plants grown under full nutrition (C, gray columns) vs sulfate deprivation (-S, black columns). Significant differences ($p < 0.05$) between -S and the respective C are represented by an asterisk (*). B: basal root sector, LR: lateral roots sector, ELR: emerging lateral roots sector, A: root apex, M: middle root sector.

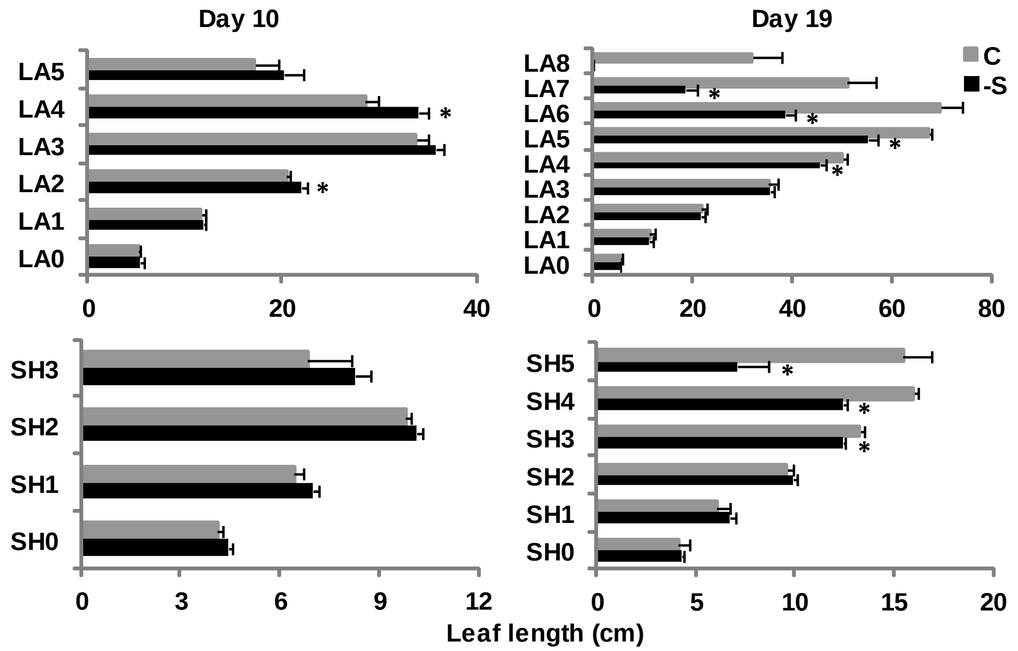

Figure S4. The length of each lamina (LA) and sheath (SH) (mean ± standard error) at days 10 and 19 of the treatment in plants grown under full nutrition (C, gray columns) vs sulfate deprivation (-S, black columns). Significant differences ($p < 0.05$) between -S and the respective C are represented by an asterisk (*).

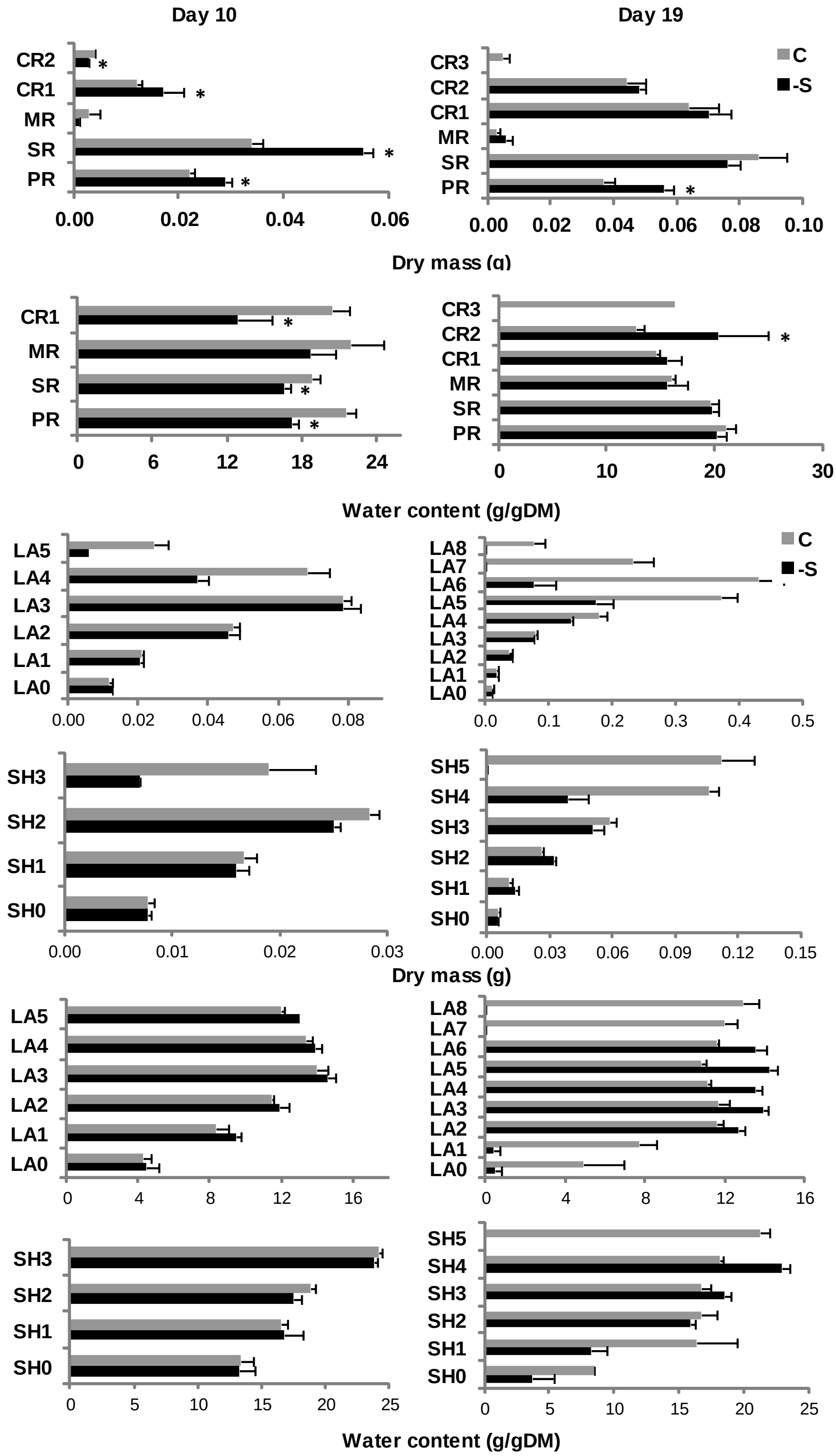

Figure S5. The allocation of the dry mass and water content in each root type and in each lamina (LA) and sheath (SH) (mean ± standard error) at days 10 and 19 of the treatment in plants grown under full nutrition (C, gray columns/ gray lines) vs sulfate deprivation (-S, black columns/ black lines). Significant differences ($p < 0.05$) between -S and the respective C are represented by an asterisk (*). PR: primary root, SR: seminal roots, MR: mesocotyl roots, CR1: 1st group of crown roots, CR2: second group of crown roots, CR3: 3rd group of crown roots.

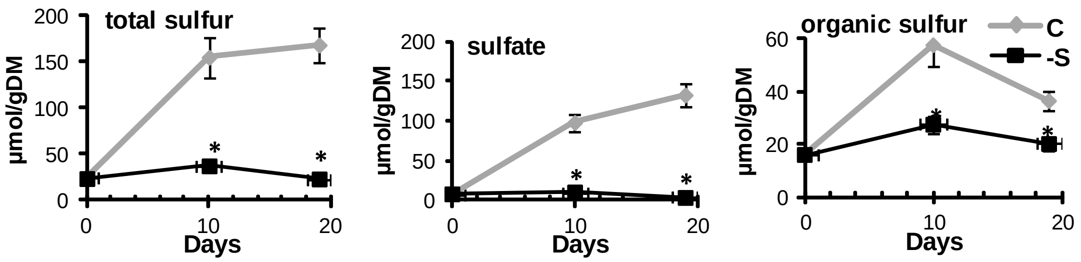

Total sulfur, sulfate and organic sulfur concentration in root system (µmol/gDM)

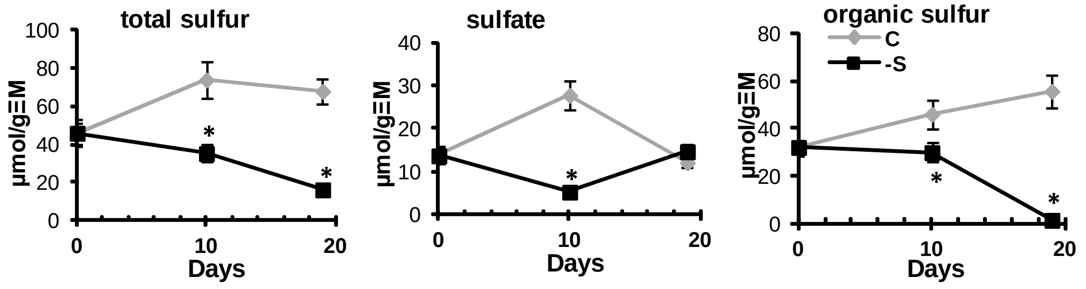

Total sulfur, sulfate and organic sulfur concentration in shoot (µmol/gDM)

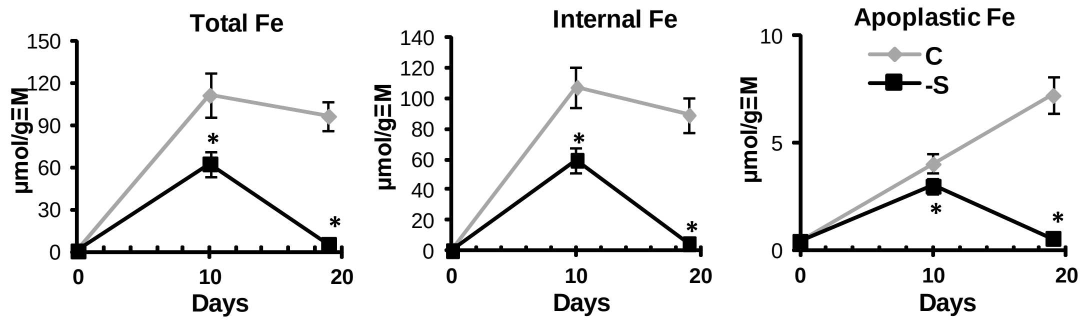

Total, internal and apoplastic iron concentration in root system (µmol/gDM)

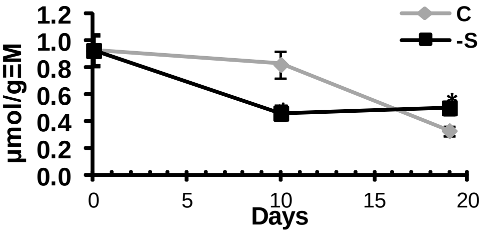

Total iron concentration in the shoot

Figure S6. The total sulfur, sulfate and organic sulfur concentration in the root system and in the aerial plant part. The total, internal and apoplastic iron concentration in the root system and in the shoot (mean ± standard error) at days 10 and 19 of the treatment in plants grown under full nutrition (C, gray columns/ gray lines) vs sulfate deprivation (-S, black columns/ black lines). Significant differences ($p < 0.05$) between -S and the respective C are represented by an asterisk (*).
